# Supplementary material for: T cell responses in repeated controlled human schistosome infection compared to natural exposure
Source: Nat Commun. 2025 Jul 24;16:6827. doi: 10.1038/s41467-025-62144-8 (PMC12290099; doi:10.1038/s41467-025-62144-8)
Supplement: Supplementary file 3 — Reporting Summary [file 41467_2025_62144_MOESM3_ESM.pdf]

nature portfolio

Corresponding author(s): Emma L. Houlder

Last updated by author(s): Jul 1, 2025

Reporting Summary

Nature Portfolio wishes to improve the reproducibility of the work that we publish. This form provides structure for consistency and transparency in reporting. For further information on Nature Portfolio policies, see our Editorial Policies and the Editorial Policy Checklist.

Statistics

For all statistical analyses, confirm that the following items are present in the figure legend, table legend, main text, or Methods section.

n/a	Confirmed	
☐	☒	The exact sample size (*n*) for each experimental group/condition, given as a discrete number and unit of measurement
☐	☒	A statement on whether measurements were taken from distinct samples or whether the same sample was measured repeatedly
☐	☒	The statistical test(s) used AND whether they are one- or two-sided<br>*Only common tests should be described solely by name; describe more complex techniques in the Methods section.*
☐	☒	A description of all covariates tested
☐	☒	A description of any assumptions or corrections, such as tests of normality and adjustment for multiple comparisons
☐	☒	A full description of the statistical parameters including central tendency (e.g. means) or other basic estimates (e.g. regression coefficient) AND variation (e.g. standard deviation) or associated estimates of uncertainty (e.g. confidence intervals)
☐	☒	For null hypothesis testing, the test statistic (e.g. *F*, *t*, *r*) with confidence intervals, effect sizes, degrees of freedom and *P* value noted<br>*Give P values as exact values whenever suitable.*
☒	☐	For Bayesian analysis, information on the choice of priors and Markov chain Monte Carlo settings
☒	☐	For hierarchical and complex designs, identification of the appropriate level for tests and full reporting of outcomes
☒	☐	Estimates of effect sizes (e.g. Cohen's *d*, Pearson's *r*), indicating how they were calculated

Our web collection on statistics for biologists contains articles on many of the points above.

Software and code

Policy information about availability of computer code

Data collection	Flow cytometry data was collected with Spectral flow version 3.1 (Cytek)
Data analysis	Processing and analysis of flow cytometry data was performed with OMIQ (Dotmatics) software, with statistical analysis performed in r. Statistical analysis and visualization was done in R (v4.3.1 core team 2023) and R studio (version 2023.06.1) R package lmerTest (version 3.1-3) was used for linear mixed models. All other analysis used package rstatix v 0.7.2. Graphs were drawn using ggplot2 (version 3.5.1).

For manuscripts utilizing custom algorithms or software that are central to the research but not yet described in published literature, software must be made available to editors and reviewers. We strongly encourage code deposition in a community repository (e.g. GitHub). See the Nature Portfolio guidelines for submitting code & software for further information.

Data

Policy information about availability of data

All manuscripts must include a data availability statement. This statement should provide the following information, where applicable:

- Accession codes, unique identifiers, or web links for publicly available datasets
- A description of any restrictions on data availability
- For clinical datasets or third party data, please ensure that the statement adheres to our policy

All data generated or analyzed during this study are included in this paper, its Supplemetary information and the Source Data file. This paper does not include any data types with mandatory deposition.

Research involving human participants, their data, or biological material

Policy information about studies with human participants or human data. See also policy information about sex, gender (identity/presentation), and sexual orientation and race, ethnicity and racism.

Reporting on sex and gender	Sex was determined by self-reporting. Sex and gender was included in study design. For endemic participants 50% of the uninfected group were male, and 33% of the infected group. For non-endemic CHI participants , 50% of the infection control group were male, and 42% of the reinfection group. All subjects were analyzed together given the small cohort size.
Reporting on race, ethnicity, or other socially relevant groupings	Socially constructed or socially relevant categorization variables such as race or ethnicity were not used in this manuscript.
Population characteristics	Endemic participants:<br>-Aged 18-25, with a median of 19 in both infected and uninfected groups.<br>-Sex, 50% of the uninfected group were male, and 33% of the infected group.<br>-All participants included in this work screened and negative for other helminth infections (Ascaris, Trichuris, Strongyloides, --Trichostrongylus, S. haematobium, or Hookworm). Participants with a history of pulmonary disease were excluded. No other co-infections were assessed.<br>-Four individuals in the uninfected group had evidence for prior S. mansoni exposure.<br>Repeated CHI participants:<br>-Aged 18-44, with a median of 24 in the infection control and 29 in the reinfection group.<br>-Sex, 50% of the infection control group were male, and 42% of the reinfection group (17).<br>-No CHI participants had a history of S. mansoni infection.
Recruitment	• Participants were recruited via advertising (posters, instagram etc) for the controlled human infection study (Netherlands).<br>• Participants were recruited in person for the endemic patent infection study (Uganda), within a 2km radius of the Kigungu landing site controls from colleges, cases from the community, with the majority of the cases doing work involving regular lake contact.<br>No clear self-selection bias is present.
Ethics oversight	The study protocols were approved by:<br>• Leiden University Medical Center Institutional Medical Ethical Research Committee<br>• Uganda Virus Research Institute Research Ethics Committee<br>• Manchester University Research Ethics Committee<br>• Ugandan National Council for Science and Technology

Note that full information on the approval of the study protocol must also be provided in the manuscript.

Field-specific reporting

Please select the one below that is the best fit for your research. If you are not sure, read the appropriate sections before making your selection.

☒ Life sciences ☐ Behavioural & social sciences ☐ Ecological, evolutionary & environmental sciences

For a reference copy of the document with all sections, see nature.com/documents/nr-reporting-summary-flat.pdf

Life sciences study design

All studies must disclose on these points even when the disclosure is negative.

Sample size	For the repeat CHI study sample size was powered in accordance with the primary outcome (infection). Specifically we calculated that 11 participants would be required in each group to detect a 70% relative reduction in CAA positivity with 80% power and (2-sided) $\alpha = 0.05$ significance level. To account for loss to follow-up, we aimed to include 24 participants, 12 in each group. Power calculations were not performed when selecting endemic sample size, as this was a pilot study.
Data exclusions	For flow cytometry analysis two (uninfected) endemic samples and four (infected) endemic samples were excluded due to low viability post-stimulation (<55% live). One repeated CHI participant in the infection control group was excluded from the analysis of schistosome-specific cytokines due to high cytokine production in the unstimulated control (>1%). Inclusion criteria was not pre-established.
Replication	PBMC samples from week 30 of the repeat CHI study were ran in two separate experiments, one to assess changes over time within the repeat CHI, and one to compare endemic to repeat CHI. Relative frequencies of the studied cytokines were comparable between these experiments, supporting the replicability of our findings (e.g. the attempt at replication was successful). Given the limited amounts of the unique sample material, we were not able to replicate the other experiments.
Randomization	Repeat CHI participants were randomized to reinfection or infection control (single infection) groups. Randomisation was not used in the endemic study, with participants assigned to groups based upon schistosome infection status. To control for covariates when randomisation was not possible (endemic study) all participants were recruited from a small area (2km) within Kigungu, Entebbe, with matched ages and sex/

gender. We were not able to control for unknown covariates such as diet or socio-economic status.

Blinding

Double-blinding was used in the repeat CHI study during data collection. Investigators were blinded for the endemic study during data collection. Blinding was not used for data analysis, with all gates (flow cytometry) set consistently for all samples, reducing the possibility of investigator bias. For luminex data blinding was not performed, but samples were randomised between plates and calculated with the same standard curve, meaning investigator bias would be minimal.

Reporting for specific materials, systems and methods

We require information from authors about some types of materials, experimental systems and methods used in many studies. Here, indicate whether each material, system or method listed is relevant to your study. If you are not sure if a list item applies to your research, read the appropriate section before selecting a response.

Materials & experimental systems

n/a	Involved in the study
☐	☒ Antibodies
☒	☐ Eukaryotic cell lines
☒	☐ Palaeontology and archaeology
☒	☐ Animals and other organisms
☐	☒ Clinical data
☒	☐ Dual use research of concern
☒	☐ Plants

Methods

n/a	Involved in the study
☒	☐ ChIP-seq
☐	☒ Flow cytometry
☒	☐ MRI-based neuroimaging

Antibodies

Antibodies used

• Extracellular: CD38 (APC-Fire, Biolegend, 1:1500, 356643), CD8 (Pacific orange, Thermofisher, 1:1000, MHCDO830), CD25 (BUV563, BD, 1:750, 612918), CD27(APC-H7, BD, 1:500, 560222), CD11b (BV510, Biolegend, 1:750, 101263), CD123 (BV510, Biolegend, 1:750, 306022), CD19 (BV605, Biolegend, 1:750, 302244), CD4 (cFlourYG584, CYTEK, 1:750, SKU R7-200), CD3 (BUV395, BD, 1:200,563546), CD27 (APC-H7, BD, 1:500, 560222), CD56 (BV510, Biolegend, 1:500, 318340), PD1 (BV750, BD, 1:375, 747446), γδTCR (BV480, BD, 1:250, 747446), CD127 (R718, BD, 1:100, 566967), CCR7 (BV785, Biolegend, 1:40, 353230), HLA-DR (PE-fire, Biolegend, 1:400, 307683) and human Fc block (Invitrogen, 1:200, 14-91613)
• Intracellular: TNF (PE-Cy7, BD, 1:3000, 557647), IL-17A (Pacific blue, Biolegend, 1:3000, 512312), IL-5 (APC, Biolegend, 1:1000, 504306), IL-4 (BUV737, BD, 1: 750, 612835), IFN-γ (BV650, BD, 1:750, 563416), IL-13 (BV711, BD, 1:750, 564288), IL-9 (PE, Biolegend, 1:500, 507605), IL-10 (PerCP ef710, Thermofisher, 1:250, 46-7108-4), CTLA (PE-Cy5, BD, 1: 10000, 555854), IL-21 (AF647, BD, 1:200, 560493), FoxP3 (PE-Dazzle594, Biolegend, 1:150, 320126), IL-22 (Vio515, Miltenyi, 1:100, 130-108-09), GATA3 (BV421, BD, 1:200, 563349), CD45RA (BUV496, BD, 1:750, 741182), CD45RA (BUV496, BD, 1:750, 741182), CD45RO (BUV805, BD, 1:750, 748367) and CRTh2 (BUV661, BD, 1:200, 741663)

Validation

Validation of antibodies was performed by manufacturers as below.

Biolegend antibodies:
• Specificity testing of 1-3 target cell types with either single- or multi-color analysis (including positive and negative cell types).
• Once specificity is confirmed, each new lot must perform with similar intensity to the in-date reference lot. Brightness (MFI) is evaluated from both positive and negative populations. Each lot product is validated by QC testing with a series of titration dilutions.

BD antibodies:
• Antibody specificity
• The specificity is confirmed using multiple methodologies that may include a combination of flow cytometry, immunofluorescence, immunohistochemistry or western blot to test staining on a combination of primary cells, cell lines or transfectant models.
• All flow cytometry reagents are titrated on the relevant positive or negative cells. To ensure consistent performance from lot-to-lot, each reagent is bottled to match the previous lot MFI.

Thermofisher(eBiosciences) antibodies:
• Target specificity validation - helps ensure the antibody will bind to the correct target. Our antibodies are being tested using at least one of the following methods to ensure proper functionality in researcher's experiments.
• Knockout—expression testing using CRISPR-Cas9 cell models
• Knockdown—expression testing using RNAi to knockdown gene of interest
• Independent antibody verification (IAV)—measurement of target expression is performed using two differentially raised antibodies recognizing the same protein target
• Cell treatment—detecting downstream events following cell treatment
• Relative expression—using naturally occurring variable expression to confirm specificity
• Neutralization—functional blocking of protein activity by antibody binding
• Peptide array—using arrays to test reactivity against known protein modifications
• SNAP-ChIF"—using SNAP-ChIP to test reactivity against known protein modifications
• Immunoprecipitation-Mass Spectrometry (IP-MS)—testing using immunoprecipitation followed by mass spectrometry to identify

antibody targets

Clinical data

Policy information about clinical studies

All manuscripts should comply with the ICMJEguidelines for publication of clinical research and a completedCONSORT checklist must be included with all submissions.

Clinical trial registration: NCT05085470

Study protocol: The full study protocol is available on request at the corresponding author.

Data collection:
- Recruitment was performed in November 2021 in Leiden, Netherlands.
- Clinical samples (including PBMCs) were taken between December 2021 and Augst 2022.

Outcomes: Primary outcome was protective efficacy and safety. Immunological outcomes were secondary outcome measures. These are defined further on https://clinicaltrials.gov/study/NCT05085470?term=recohsi&rank=1.

Plants

Seed stocks: *Report on the source of all seed stocks or other plant material used. If applicable, state the seed stock centre and catalogue number. If plant specimens were collected from the field, describe the collection location, date and sampling procedures.*

Novel plant genotypes: *Describe the methods by which all novel plant genotypes were produced. This includes those generated by transgenic approaches, gene editing, chemical/radiation-based mutagenesis and hybridization. For transgenic lines, describe the transformation method, the number of independent lines analyzed and the generation upon which experiments were performed. For gene-edited lines, describe the editor used, the endogenous sequence targeted for editing, the targeting guide RNA sequence (if applicable) and how the editor was applied.*

Authentication: *Describe any authentication procedures for each seed stock used or novel genotype generated. Describe any experiments used to assess the effect of a mutation and, where applicable, how potential secondary effects (e.g. second site T-DNA insertions, mosiacism, off-target gene editing) were examined.*

Flow Cytometry

Plots

Confirm that:

- [x] The axis labels state the marker and fluorochrome used (e.g. CD4-FITC).
- [x] The axis scales are clearly visible. Include numbers along axes only for bottom left plot of group (a 'group' is an analysis of identical markers).
- [x] All plots are contour plots with outliers or pseudocolor plots.
- [x] A numerical value for number of cells or percentage (with statistics) is provided.

Methodology

Sample preparation:

PBMC isolation, storage, and thawing

PBMC isolation from endemic-infection blood samples was performed at UVRI, Uganda, while those from the repeated CHI were isolated at LUMC, Netherlands both using the Ficoll gradient density separation method. In summary, venous whole blood samples collected in heparin tubes were diluted in HBBS (Invitrogen) and separated over a Ficoll gradient (Apotheek LUMC) by centrifugation at low break at 400g, at room temperature for 25 minutes.. PBMCs collected were then washed with HBSS, counted, and cryopreserved in complete RPMI (Invitrogen), with 100 U/ml penicillin G sodium, 100 µg/ml streptomycin (Sigma), 1 mM pyruvate (Sigma), and 2 mM glutamine (Sigma); with 10% DMSO (Merck) and 20% FCS (Bodinco). Cryopreserved cells were stored at -80oC overnight and transferred to liquid nitrogen for long-term storage. PBMC isolation and cryopreservation at UVRI followed the same protocol except for the following differences: all washes were in complete RPMI, centrifugation by Ficoll gradient at 1000g for 22 minutes, and cryopreservation used a higher FCS percentage (50%).

Cryopreserved PBMC samples from Uganda were transported to the LUMC and thawed together with frozen PBMCs from the ReCoHSI trial. Thawing was performed at 37oC in thawing media (complete RPMI 1640, 20% FCS, benzonase 25 units/ml (Merck).

Stimulation for cytokine production

Up to 1x106 cells per condition were plated into culture media (complete RPMI and 10% FCS) in round-bottom well plates (BD Biosciences) and stimulated with culture media, SEA (10 µg/ml), CA (50 µg/ml) and AWA (50 µg/ml) for 24hrs at 37oC

	incubation. Four hours after the beginning of incubation, Brefeldin A (5mg/ml, Sigma) was added to wells and then incubated for a further 20 hours. Upon completion of stimulation, cells were centrifuged (400xg, for 4 min) in a V-bottom plate (BD Biosciences) and supernatant transferred to 96-well round-bottomed plates (BD Biosciences) for flow cytometry staining.
Instrument	Cytek Aurora 5L (Cytek Biosciences, Fremont, CA, USA)
Software	Flow cytometry data was collected with Spectral flow version 3.1 (Cytek). Processing and analysis of flow cytometry data was performed with OMIQ (Dotmatics) software.
Cell population abundance	Not applicable – cell sorting was not performed. Cell populations are shown in graphs
Gating strategy	For all samples initial an initial 'time' gate was used in which viability was placed against time and a period of stable acquisition was chosen. Singlets were then chosen based upon FSCH vs FSC-A. Lymphocytes were then gated based on SSC-A and FSC-A. Live cells were then gated based upon Viability vs FSC-A. Next, myeloid cells, basophils, B cells and NK cells were gated out. T cells were then gated as CD3+. TCRgd was used to gate out gd T cells. Then CD4 and CD8+ cells were selected. T cell subpopulations were gated using CD45RA, CCR7, Foxp3, CD25, CD38 and CD27. For cytokines, gates were set using the medium control of fluorescence minus many controls.

☒ Tick this box to confirm that a figure exemplifying the gating strategy is provided in the Supplementary Information.
